# Gaps and challenges in the provision of treatment for patients with end-stage renal disease: perspectives from the Philippines

**DOI:** 10.1016/j.lanwpc.2023.100889

**Published:** 2023-08-31

**Authors:** Janine Audrei T. Pajimna, Giannina Alyana L. Orpilla, Mark Jason D.C. Milan, Cassandra Tria S. Virtucio, Joseph Virgilio M. Pamatian

**Affiliations:** a Department of Medicine, St. Luke’s Medical Center-Quezon City, 279 E Rodriguez Sr. Ave, Quezon City, Metro Manila 1112, Philippines; b University of Santo Tomas, España Boulevard, Sampaloc, Manila, Metro Manila 1008, Philippines; c Department of Pediatrics, University of the Philippines-Philippine General Hospital, Taft Avenue, Ermita, Manila, Metro Manila 1000, Philippines; d Far Eastern University-Nicanor Reyes Medical Foundation, Regalado Avenue, West Fairview, Quezon City, Metro Manila 1118, Philippines

Chronic kidney disease (CKD) is a global public health concern, with prevalence of 9.1%–13.4% of the population worldwide.^1^ In the Philippines, its prevalence is 35.94%, which is much higher than estimated global rates.^2^ Aside from its contribution to mortality, the growing burden of CKD is also illustrated by its associated financial costs. Locally, 94% of end stage renal disease (ESRD) patients are undergoing center-based hemodialysis (HD), 4% are on peritoneal dialysis (PD) and only 2% had kidney transplantation (KT). Despite KT being the gold standard treatment for ESRD, HD is still preferred by most Filipino patients due to transplant costs, low organ donations, lack of capable infrastructures, and long term immunosuppression therapy.^3^

The Philippines depends on the national health insurance program, PhilHealth, which ideally guarantees all citizens automatic enrollment pursuant to the Universal Health Care Law of 2019. However, data of registered beneficiaries from 2018 to 2021 observed geographical discrepancies, with a few remote provinces noted to have coverages of only 52% and below.^4^ There are also some gaps in provision of financial assistance to the beneficiaries. PhilHealth covers the costs of HD, PD and KT in varying degrees. However, most patients still choose HD over PD, despite better coverage of PD in the past.^3^ It was only in June 2023 when PhilHealth's coverage was expanded to 156 HD sessions to fully cover the recommended thrice-weekly annual treatments. This is an improvement from the 45, 90, and 144 HD sessions previously amended in years 2013, 2015, and 2020, respectively, wherein patients had to resort to twice-weekly sessions or pay out-of-pocket.

In general, preference for center-based HD treatments are due to supervised care,^5^ patient burnout, family burden, and lack of confidence in self-treatment.^6^ However, one strategy to minimize the healthcare workforce strain and to reduce the economic costs of ESRD is increasing the use of home-based dialysis modalities, be it PD or home-based HD.^6^ HD centers are also more concentrated in urbanized cities, hence home-based treatments would be ideal for the Philippines, where geographical access for healthcare is a problem, especially in rural areas.^3^ Advantages of home-based dialysis also include lower costs from manpower and infrastructures^7^ and less patient infection exposure.^8^

Home-based HD is a modality that has already been rising worldwide, especially in higher-income countries like New Zealand with 52% of patients utilizing the modality as of 2016.^7^ It is correlated with improved cardiovascular, nutritional and quality of life parameters, as well as long-term survival advantages. Despite this, it is still not yet widely adopted in low- and middle income countries (LMIC) like the Philippines. Although this can be seen as a lost opportunity to improve the quality adjusted survival rates of patients, implementing this modality would be a challenge in an LMIC such as the Philippines, since it is currently not covered by health insurance and there are no reimbursement policies for its utilization yet. Patients from lower socioeconomic classes also might not be eligible to use this modality due to their poor housing conditions.

In 2014, PhilHealth introduced a PD-First Z-Benefit Package, which promotes PD as the first line modality for eligible ESRD patients. It allows for coverage of up to 90–120 PD solutions per month or three to four PD solutions per day. Although in present practice, only three bags per day are actually covered, and those with prescriptions requiring more are either under dialyzed or have to pay out-of-pocket.

PD is underutilized in the country despite the implementation of the above policy. To date, no local studies were done to explore the reasons why more Filipinos opt for HD over PD. Studies done in Southeast Asia identified that patients’ unwelcoming attitudes towards PD arise from many factors such as fear of peritonitis, daily versus thrice-weekly treatments, and lack of education about the modality.^5^ A study done in India also demonstrated that physicians’ recommendations strongly influenced patients’ choice of renal replacement therapy modality.^9^ Physician’s bias for HD versus PD are affected not only by medical contraindications but also by lack of formal training, specialized centers,^5^^,^^6^ and incentives for using the modality.^6^

All these said, the increased utilization of home-based modalities, specifically PD, could potentially relieve financial burdens associated with ESRD in the Philippines. However, the existing biases of both healthcare workers and patients against PD is an end-effect of lack of infrastructures, manpower, and financial support for the modality. It is perpetuated by the vicious cycle of lack of enrollees, less demand and hence, less funding to fully operationalize the PD-First system in the country.

## Contributors

JAP and GAO conceptualized the idea and wrote the original draft of the manuscript. JAP, GAO, MJM, CTV and JVP reviewed and revised the manuscript.

## Declaration of interests

The authors declare no competing financial interests or personal relationships that could have appeared to influence the work reported in this paper.
